# Institutional capacity for health systems research in East and Central African schools of public health: experiences with a capacity assessment tool

**DOI:** 10.1186/1478-4505-12-21

**Published:** 2014-06-02

**Authors:** Nasreen Jessani, Daniela Lewy, Elizabeth Ekirapa-Kiracho, Sara Bennett

**Affiliations:** 1 Department of International Health, Johns Hopkins Bloomberg School of Public Health, 615 North Wolfe Street, Baltimore, MD 21205, USA; 2 Department of Health Policy Planning and Management, Makerere University School of Public Health, P.O. Box 7072, Kampala, Uganda

**Keywords:** Capacity assessment, Central Africa, East Africa, Health systems research, Schools of public health

## Abstract

**Background:**

Despite significant investments in health systems research (HSR) capacity development, there is a dearth of information regarding how to assess HSR capacity. An alliance of schools of public health (SPHs) in East and Central Africa developed a tool for the self-assessment of HSR capacity with the aim of producing institutional capacity development plans.

**Methods:**

Between June and November 2011, seven SPHs across the Democratic Republic of Congo, Ethiopia, Kenya, Rwanda, Tanzania, and Uganda implemented this co-created tool. The objectives of the institutional assessments were to assess existing capacities for HSR and to develop capacity development plans to address prioritized gaps. A mixed-method approach was employed consisting of document analysis, self-assessment questionnaires, in-depth interviews, and institutional dialogues aimed at capturing individual perceptions of institutional leadership, collective HSR skills, knowledge translation, and faculty incentives to engage in HSR. Implementation strategies for the capacity assessment varied across the SPHs. This paper reports findings from semi-structured interviews with focal persons from each SPH, to reflect on the process used at each SPH to execute the institutional assessments as well as the perceived strengths and weaknesses of the assessment process.

**Results:**

The assessment tool was robust enough to be utilized in its entirety across all seven SPHs resulting in a thorough HSR capacity assessment and a capacity development plan for each SPH. Successful implementation of the capacity assessment exercises depended on four factors: (i) support from senior leadership and collaborators, (ii) a common understanding of HSR, (iii) adequate human and financial resources for the exercise, and (iv) availability of data. Methods of extracting information from the results of the assessments, however, were tailored to the unique objectives of each SPH.

**Conclusions:**

This institutional HSR capacity assessment tool and the process for its utilization may be valuable for any SPH. The self-assessments, as well as interviews with external stakeholders, provided diverse sources of input and galvanized interest around HSR at multiple levels.

## Background

Since the early 2000s there has been increasing recognition of and support for health systems research (HSR) as a means to strengthen health policies, programs, and practices in low- and middle-income countries [1,2]. It is widely acknowledged, however, that one of the barriers to more effective generation and use of health systems evidence is the limited capacity for HSR in many countries, particularly in low-income countries [3,4]. A lack of capacity for HSR may manifest itself in multiple ways, such as an insufficient number of skilled researchers to conduct multidisciplinary research, inadequate library or information systems, unsupportive organizational environments, or an absence of linkages to policymakers and decision-makers who form the principal audience for much HSR. Given this array of challenges, it can be difficult to know where to start in terms of building capacity for HSR.

Despite significant investments in research capacity development by agencies [5,6] such as the Wellcome Trust, the Fogarty International Center, Canada’s International Development Research Centre (IDRC), and the Swedish International Development Cooperation Agency, among others, there is a strikingly limited literature on assessing research capacity [7]. Frameworks for assessing health research capacity strengthening exist; however, many of these have been designed to satisfy funder’s needs and few provide practical guidance for implementation of the frameworks [8]. Furthermore, systematic methods of assessing existing capacity as an input to identifying areas for strengthening were not available. Commonly employed approaches have only been used retrospectively [9] and, typically, have focused on easily measured quantitative indicators of research and training outputs, such as the number of people trained and number of articles published [10,11], rather than less easily measured but equally important outcomes such as changes in organizational culture or attitudes. Furthermore, with only a few notable exceptions [10,12-14], very little of this literature has focused on HSR.

This paper reports on an initiative supported by the Future Health Systems consortium [15] through the Higher Education Alliance for Leadership Through Health (HEALTH), which is a consortium of seven schools of public health (SPHs) in East and Central Africa. The SPHs included are:

a) Jimma University College of Public Health and Medical Sciences (CPHMS), Ethiopia;

b) Kinshasa School of Public Health, (KSPH), Democratic Republic of the Congo (DRC);

c) Makerere University College of Health Sciences School of Public Health (MakSPH), Uganda;

d) Moi University School of Public Health (MUSOPH), Kenya;

e) Muhimbili School of Public Health and Social Sciences (MUSPHSS), Tanzania;

f) National University of Rwanda School of Public Health (NURSPH), Rwanda;

g) University of Nairobi School of Public Health (SPHUoN), Kenya.

Brief details about each of the seven schools are listed in Table 1. Among the seven SPHs, only KSPH, DRC, had previously undergone an organizational assessment that included the identification of public health training needs and characterization of faculty capacity to teach public health [16]. However, the scope was rather wide, the evaluation was externally contracted, and the study did not include HSR. Similarly, in Uganda, a 2010 assessment encompassed the entire College of Heath Sciences and its contribution to improving health in the country [17]. Hence, the overarching aim of this initiative was to devise self-led organizational HSR capacity assessments that would result in internally cultivated HSR capacity development strategies for the HEALTH Alliance SPHs.

**Table 1:** Summary of each school of public health capacity self-assessment process

	**Year established**	**No. of faculty**	**Self-administered section of assessment tool**		**Key informant interviews**	
			**No. of respondents**	**Type of respondents**	**No. of respondents**	**Type of respondents**
Jimma University College of Public Health and Medical Science, Ethiopia (CPHMS, Ethiopia)	2009	285	26	Purposive selection based on seniority: office holders (department heads) and relevance to research (research office holders)	10 internal	Faculty, senior management
	
					6 external	Ministry of Health
						World Health Organization
						Oromiya Regional Health Bureau
						JHPIEGO
						EHNRI
						EPHA
Kinshasa School of Public Health, DRC (KSPH, DRC)	1985	38	35	Professors, Head of departments, lecturers	8 internal	Faculty
					26 external	13 – Ministry of Health senior officers
						9 – Donor representatives
						4 – National NGO officers
Makerere University College of Health Sciences School of Public Health, Uganda (MakSPH, Uganda)	1974	58	15	Full time SPH faculty only. No part time lecturers from NGOs or MOH	1 internal	Faculty
	
					6 external	2 – Members of parliament
						2 – District health officers
						1 – Desk officer Ministry of Gender
						1 – Executive Director at Uganda Catholic Medical Bureau
Moi University School of Public Health, Kenya (MUSOPH, Kenya)	1997	35	22	College of Health Sciences Faculty – Schools of Medicine, Nursing, Dentistry and Public Health	6 internal	Dean
						Deputy Vice Chancellor
						2 – AMPATH
						2 – Moi Teaching and Referral Hospital (MTRH)

					15 external	6 – Ministry of Health (National and District levels)
						2 – National Teaching and Referral Hospitals (Head research and Ag. deputy director)
						3 – Development partners (WHO, MSH and JICA)
						2 – Universities (GLUK and University of Nairobi)
						1 – NGOs (AMREF)
						1 – Research institution (KEMRI)
Muhimbili School of Public Health, Tanzania (MUSPHSS, Tanzania)	1991	43	16	SPH faculty (Junior and senior, fulltime)	1 internal	SPH-Dean
		
					4 external	2 – MoH (Director for Policy and Planning and Head of HSR Unit)
						1 – National Institute of Medical Research (NIMR) Director of Research
						1 – World Health Organization
National University of Rwanda School of Public Health, Rwanda (NURSPH, Rwanda)	2000	19	4	SPH faculty	4 external	1 – US Centers for Disease control (CDC)
						1 – Belgian Technical Corporation
						1 – MoH (Acting head of Maternal and Child Health)
						1 – MoH (Head of Research and planning)
University of Nairobi School of Public Health, Kenya (SPHUoN, Kenya)	2010	18	5	Faculty who teach some aspect of health services management, policy, health economics, primary healthcare	16 internal	4 – Leadership (Director, SPH and thematic heads)
						12 – Junior and senior faculty
		
					12 external	1 – Ministry of Medical Services
					(Accessed easily due to attendance at a leadership and health services management course)	1 – Ministry of Public Health and Sanitation
						3 – Universities (Great Lakes University, Strathmore University)
						4 – Development partners (World Bank, JICA, MSH, GIZ)
						2 – National Medical Training and Referral Centers (Kenyatta National Hospital, Kenya Medical training college)
						1 – Institute of Health Policy Management and Research

As the aim of the assessment was primarily to identify where capacity development investments in HSR would have the greatest impact, a narrow focus on outputs was inappropriate. Our review of the literature found only one other published paper that shared a similar objective, employing a capacity assessment tool that combined both quantitative and qualitative elements, to help strengthen and shape a capacity development intervention; however, the study was focused on a single training intervention and did not concern HSR [18]. Since the completion of the HEALTH Alliance exercise, there has been one other relevant publication [19]. Its thrust, however, is on the outcomes, not the process, of a capacity assessment for health policy and systems research and analysis.

Past initiatives in sub-Saharan Africa have focused primarily on identifying and enhancing individual competencies [20-22], but the institutional environments within which individuals work are often key to retaining and empowering them [23,24]. Our approach was to develop a capacity assessment process that could provide a systematic method for each of the SPHs to reflect on institutional strengths and weaknesses with respect to HSR. The capacity assessment tool also aimed to stimulate discussion and agreement on what strategies would be most effective to help develop HSR capacity. Note that the focus of the assessment was organizational capacity for HSR, although individual researcher capacity was also addressed.

The main objectives for the capacity assessment process were the following.

• Through a process of self assessment, develop a clear picture of existing capacities for HSR at the HEALTH Alliance SPHs.

• For each SPH, draft and build consensus around HSR capacity development strategies and work plans for implementing them.

• Make an initial and rapid assessment of HSR priorities in the different countries involved in the HEALTH Alliance, in part as a means to strengthen links to the policymaking and decision-making community.

While the primary purpose of the assessment was not to provide a “baseline” against which changes in capacity could be assessed, some of the indicators captured may be feasible to use in this way. Findings from the self-assessments are reported separately [25-27]. This paper reviews the design and application of the self-assessment tool, the resulting outcomes, and lessons learned through its implementation with the aim of informing others who seek to use similar tools.

The following section presents the self-assessment tool in more detail. We then briefly describe the methods used to review and reflect on experience with the self-assessment tool. The findings section presents (i) the actual capacity assessment process and how it varied across countries, (ii) the key outcomes of the assessment, and (iii) the factors that affected the assessment process.

## Methods

### The capacity assessment tool

The overall structure of the tool was derived primarily from an instrument that IDRC uses to assess the organizational capacity needs of its partner research organizations [28], but with questions adapted to focus specifically on HSR. Some specific questions were informed by a qualitative instrument that was used to assess health policy analysis institutes, including their capacity and sustainability [29], and by tools used by the Alliance for Health Policy and Systems Research [4]. The proposed implementation process and format of the tool drew upon processes recommended in a similar tool developed by the Canadian Health Service Research Foundation (CHSRF) that seeks to examine the capacity of organizations to acquire and apply research evidence [30]. The CHSRF tool combines individual self-assessments with discussions across the organization that aim to stimulate action to address identified weaknesses.

A first draft of the capacity assessment tool was developed by Johns Hopkins University (JHU) in early 2011. The Deans of the SPHs identified focal persons (FPs) to lead the initiative, and this group together with JHU colleagues met at a workshop in Uganda in June 2011, where the tool was revised and adapted to meet local needs. The tool consisted of three components described below, designed to provide both internal and external perspectives, as well as subjective and objective information on capacity. The majority of amendments made during the June 2011 workshop were in the order, phrasing, and clarification of the questions. The necessity for ethics approval was discussed across the FPs with a final conclusion that all SPHs will seek clearance from their institutional Ethics Review Committees. KSPH colleagues translated the tool into French for use in the DRC. The final version of the HSR capacity assessment tool is as a separate document (Additional file 1).

The final Capacity Assessment Tool consisted of the following components:

***A: Self-assessment of existing capacity for health systems research.*** Faculty and staff who were currently or potentially engaged in HSR were asked to complete the subjective self-administered questionnaire that sought their opinions on HSR capacity at their organization. The questionnaire began by providing a definition of HSR and offering examples of studies we believe reflect HSR as well as those that do not. The instrument included questions on the external environment, organizational motivation and commitment to HSR, organizational capacity (including the relevant skills of individual faculty and staff), research support, and inter-institutional linkages. There were multiple items regarding capacity under each of these headings, and respondents used a 5-point Likert scale (1 = strongly disagree, 5 = strongly agree) to indicate the extent to which they agreed with each statement. The data was captured on paper and transferred to electronic Microsoft Excel format. Average scores per item were calculated as described elsewhere [25]. Table 2 provides examples of statements that respondents rated.

**Table 2:** Illustrative questions from self-administered internal faculty survey

	**1**	**2**	**3**	**4**	**5**
	**Strongly disagree**	**Disagree**	**Neither agree nor disagree**	**Agree**	**Strongly agree**
***Forces in the external environment***					
**A.2.1** Key institutions in this country, such at the Ministry of Health, have a strong interest in health systems					
**A.2.2** There are adequate possible funding opportunities for health systems research (HSR) at the moment					
**A.2.3** There are adequate organizations in this country which are interested in using HSR findings					
***Organizational motivation and commitment***					
**A.2.4** Our School of Public Health (SPH) places a high priority on the conduct of original research					
**A.2.5** Our SPH places a high priority on HSR					
***Organizational capacity***					
**A.2.6** I feel confident that there are individuals in this SPH who can provide high level leadership for HSR					
**A.2.7** There are an adequate number of researchers in this SPH who are interested in HSR					
**A.2.8** There are adequate library resources in this SPH to support HSR					

***B and C: Profile of health systems research within the institution.*** The first form, completed by each FP or their team, captured objective data about the number of staff working in HSR, their relevant skills, and the type of work they conduct. The second involved interviews with key informants within senior management at the institution.

***D: Identification of priority health systems research questions.*** Researchers at the SPH and key stakeholders within the country were consulted (in a “quick and dirty” exercise) to determine what the priority HSR questions are in the health systems field. In each country, the FP or their team conducted five to eight semi-structured interviews using a provided interview guide.

Once these three steps were completed, FPs and their teams conducted preliminary analyses on the results. They then sought to convene two workshops with key stakeholders from within their respective SPHs. The first of these workshops focused on discussing the emerging findings from the HSR capacity assessment, interpreting these findings, and reaching agreement on strengths and weaknesses in existing HSR capacity. The second workshop was intended to discuss and build the capacity development plan. JHU provided draft agendas for these workshops. The capacity assessment teams incorporated discussions from the first workshop into the capacity assessment report, and findings from the second workshop formed the basis of the capacity development plan. Table 3 provides an overview of the workshops held by each SPH.

**Table 3:** Summary of each school of public health’s institutional capacity assessment results dissemination strategies

	**Dissemination workshop(s)**			
	**Objective(s)**	**No. of participants**	**Type of participants**	**Comments**
**CPHMS, Ethiopia**	Workshop 1: To discuss findings, strengths, gaps, priorities identified, etc., followed by discussions on solutions and strategies for capacity development	14	Leadership and management, senior staff, and faculty	CPHMS, Ethiopia, followed the content and structure of the workshop guidelines but adapted the time allocations to each section to meet the needs of the participants
**KSPH, DRC**	Journal club: For HSR awareness raising	15	Journal club: Senior and junior faculty	This journal club was deemed important prior to the assessment exercise due to low levels of understanding of HSR
	Workshop 1: Results dissemination	10	Workshop 1: Faculty who commented on the results of the assessment about one week after writing the report	The workshop followed the guideline’s content but durations for sessions were modified to meet time schedule of participants. A brief report was sent to main MOH officers especially from Directorate of Studies and Planning with whom the team discussed research priorities and main health sector challenges
	Workshop 2: Results dissemination and action plan development	18	Workshop 2: Pedagogical council, decision makers, and all staff one week after the first workshop	
**MakSPH, Uganda**	Workshop 1: Develop priority setting agenda; results dissemination	10	Workshop 1: Respondents of the self-assessments	Attendance varied throughout the session as participants attended to other commitments
**MUSOPH, Kenya**	Workshop 1: Results dissemination	7	Workshop 1: Respondents of the self-assessments including senior colleagues and heads of departments	The meetings took about 3.5 hrs each due to the pre-arranged structure but due to the small turnout, group discussions were modified into brainstorming sessions for how the SPH could address its capacity gaps.
	Workshop 2: Continue awareness-raising in conjunction with results sharing	9	Workshop 2: All of the College of Health sciences	
**MUSPHSS, Tanzania**	Workshop 1: Collective facilitated tool administration	16	Workshop 1: Faculty targeted to complete the self-assessments	The self-assessment tool was first distributed to a small number of faculty working on HSR who then broadened the definition that the HEALTH Alliance had chosen. An initial group of self-assessment respondents were convened for sensitization purposes. After posing the questions, giving comments, discussing, and voting, the group selected a response. The FPs documented the collective responses
	Workshop 2: Results dissemination and action plan development	20	Workshop 2: All stakeholders (internal and external)	
**NURSPH, Rwanda**	Workshop 1:	9	Workshop 1:	Once the draft report was written, the FPs sent an email (with a summary of the report) one week in advance to invite faculty/staff to attend a dissemination meeting
	Discuss findings, strengths, gaps, priority setting, and results dissemination		Junior and senior faculty	
**SPHUoN, Kenya**	Workshop 1: Results dissemination and discussion of findings	22	Workshop 1: Respondents of the self-assessments (internal and external). External stakeholders (7) e.g., donors and MOH	Approx 3 hrs. Main outcome was decisions on expansion of SPHUoN visibility and collaborations
	Workshop 2: HSR capacity development priorities	24	Workshop 2: Faculty (12) and students (12)	Approx 3 hrs. The meeting focused on supporting greater capacity to write and produce outputs for priority research

**Figure 1:**
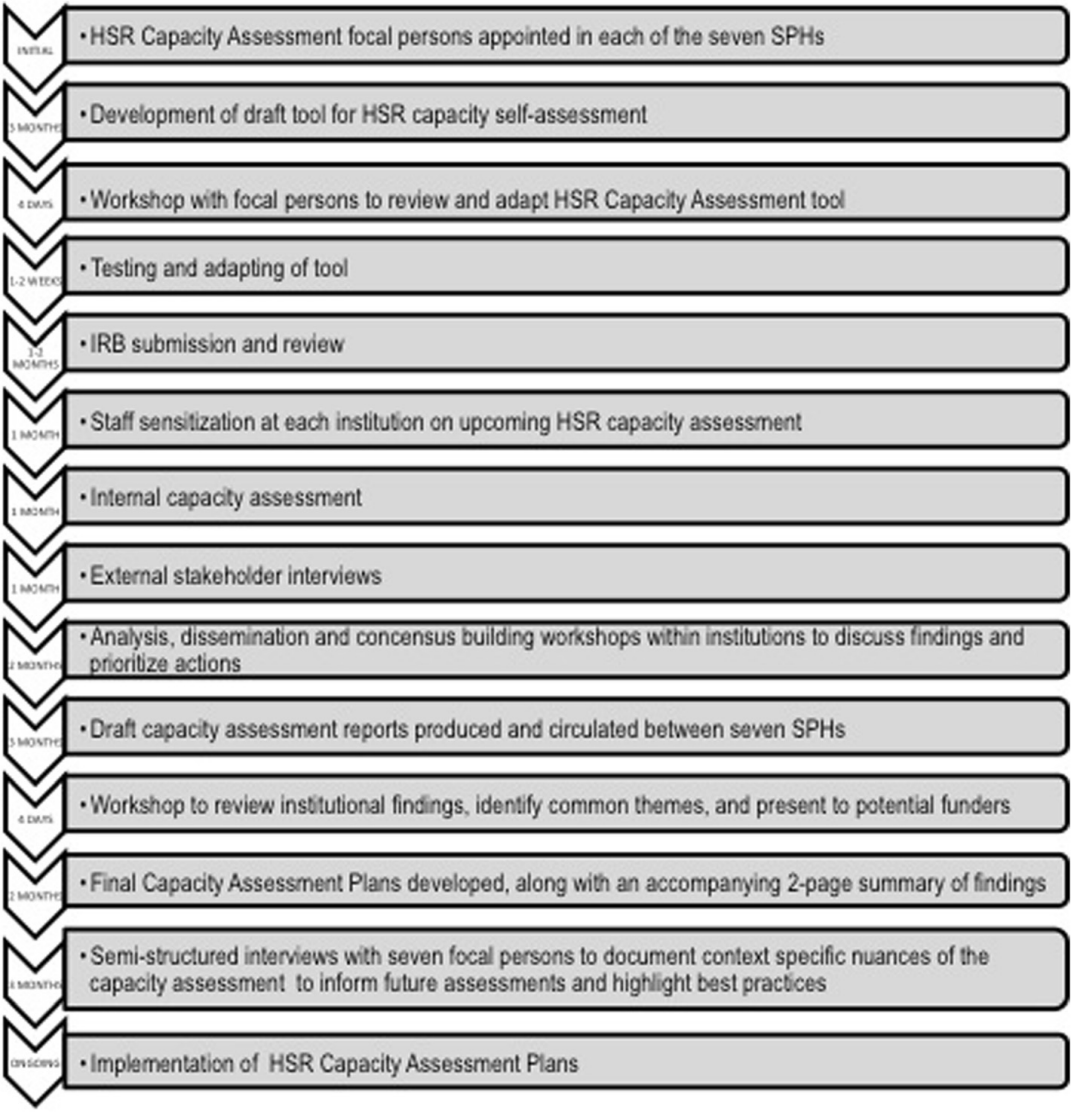
Self-assessment process – an idealized step-by-step flowchart.

The seven SPHs implemented the capacity assessment during the months that followed the June 2011 meeting. Ethics approval was sought at all sites except MUSPHSS, Tanzania, where it was deemed unnecessary by the Dean. The FPs along with JHU reconvened in December 2011 to share their results, reflect on the outcomes, and discuss priorities for capacity development plans. Each SPH created a final report and a presentation on the detailed capacity self-assessment results that were shared internally. A two-page brief of the results and priorities was produced for external audiences. Refer to Figure 1 for a flowchart of the assessment process and Table 1 for a summary of the respondents.

### Analyzing the experience of applying the capacity assessment tool

The authors of this paper tracked the implementation of the assessment tool through participating in each of the international meetings, regular teleconferences where progress was shared, and responding to occasional emails from FPs. The authors also reviewed draft findings from the institutional reports.

In addition, semi-structured interviews with all seven of the FPs in the different SPHs were conducted between March and May 2012 in order to document the differences in implementation processes and outcomes. Interview questions addressed (i) the implementation process, (ii) factors that facilitated implementation, (iii) challenges in implementation, (iv) perspectives on outcomes, and (v) advice and further thoughts. In particular, questions focused on data collection steps, deviations from protocol, human and financial resources needed for the assessment, internal dissemination of workshop details, feedback on assessment from participants and senior leadership, assessment successes, assessment challenges and solutions, reflections on outcomes, and best practices to inform future assessments. Responses were captured verbatim and extensive notes were taken during the interviews, all of which were reviewed and verified for content by respondents. Two members of the study team together reviewed the notes using a grounded theory approach [31] to generate a list of recurring themes related to ‘factors’ – both positive and negative – that affected the capacity assessment process. Similarly, they created list of ‘outcomes’ for both expected as well as unanticipated; themes were reviewed, collated, and summarized.

## Findings

### Capacity assessment process

All SPHs except for NURSPH, Rwanda, used the same capacity assessment tool. NURSPH, Rwanda, had launched the assessment in advance of the June 2011 meeting in Uganda and therefore used a previous version of the tool. A common criticism was that the tool was too long; hence, during the implementation process, each SPH made minor amendments to the tool based on their local needs. Ethics approval was sought at all sites except MUSPHSS, Tanzania, where the Dean considered the activity to be a capacity building exercise rather than a research initiative.

Except for MUSPHSS, Tanzania, the self-assessment component was sent to respondents via email as well as in hard copy for them to fill out individually and return anonymously. Respondents were given between one week and one month to return their forms. The return rates varied greatly between schools and in all cases respondents had to be reminded several times. To address low response rates, the assessment team at MUSPHSS, Tanzania, invited all of the respondents to convene together. They completed the self-assessments individually, discussed them as a group, and sought final answers through consensus. Although the assessment was designed to be self-administered, four out of seven teams (MUSOPH (Kenya), KSPH (DRC), SPHUoN (Kenya), MUSPHSS (Tanzania)) resorted to interviewer administered assessments in instances where familiarity with the objective was low, time was constrained, help was sought, or language was a barrier.

In order to complete all sections of the protocol within the budgeted time, FPs recruited assistance, particularly for pursuing respondents, collecting the completed forms, facilitating self-assessments, travelling to other cities to speak to external partners, planning dissemination meetings, data analysis, and report writing. The assessment team was made up of 3 to 5 people depending on the size of the school. Teams were varied and comprised of faculty with experience in interviewing and research skills, post-graduate students under training, external partners interested in HSR, and, in some cases, translators and transcribers. A sound understanding of HSR was a prerequisite in all cases, to ensure that the assessment team could serve as a resource for respondents in the event that there were questions or clarifications during administration of the tool.

All SPHs convened at least one workshop with the intent to share the findings of the capacity assessment. Capacity development planning was absorbed into the first dissemination workshop for CPHMS, Ethiopia, and for MakSPH, Uganda. However, it was the primary topic for the second workshop for SPHUoN, Kenya, for MUSPHSS, Tanzania, and for KSPH, DRC. NURSPH, Rwanda, did not convene a second workshop. The guidelines for the result dissemination workshops were used without significant adaptation, although the time allocation to each section of the workshop was often reduced and the method of interaction varied as a result (Table 3). The workshops lasted from 1.5 to 3 hours. In most instances the FPs facilitated the workshops except for the ones in Kinshasa, which were facilitated by the Director of KSPH, DRC. At MUSOPH, Kenya, results of the assessment were also shared online with all staff including the Dean. As shown in Table 3, all dissemination workshops involved respondents as well as key decision makers.

Reconciling the results from the various sections of the tool proved challenging. Triangulation therefore allowed for perceptions to be reinforced or for misperceptions to be amended. For instance, at MUSPHSS, Tanzania, some faculty were unaware of services and resources such as databases and library access and, assuming they did not exist, rated these as low in the self-assessment. Similarly at MUSOPH, Kenya, faculty were under the impression that organizational policies were not available. At KHSPH, DRC, approximately 60% of interviewees agreed that the SPH places high priority on HSR and 70% agreed that KSPH has an adequate number of individuals with strong quantitative research skills who are interested in applying them to HSR, but when few, if any, HSR outputs were identified, these views were called into question. Discussions and debates in the workshops led to a realization that HSR was poorly understood in the case of KSPH, DRC, and that resources are often available but either not distributed or not actively accessed in the example of MUSPSS, Tanzania, and MUSOPH, Kenya.

### Factors affecting the capacity assessment process

Four overarching factors influenced the nature and outcomes of the assessment process.

#### Leadership and collaboration

Several SPHs stressed the importance of support from senior leadership, particularly the Deans, as a key facilitator of the process. Furthermore, they mentioned that the technical and moral support from the HEALTH Alliance Africa Hub and JHU was critical, particularly during moments of delays, frustrations, and bureaucratic red tape. Building in room for flexibility in the application of tools as well as the timing allowed for a more rigorous process and an opportunity to document any deviations from protocol for learning purposes.

#### Conceptual understanding of HSR

A universal challenge centered on the term HSR. SPH faculty that do not engage much in HSR were usually unfamiliar with what HSR comprises. Thus, FPs had to explain to each person individually during the self-assessments what HSR is, assist them in determining if they are currently engaged or interested in engaging in HSR, and whether faculty research was eligible for consideration under the HSR label.

“*The lack of knowledge on HSR was overcome by explaining the operational definition by the hub that was created* [by the HEALTH Alliance] *in June 2011. Other times, HSR published papers were used as examples.*” (FP, CPHMS, Ethiopia)

The lack of understanding of HSR in general affected the number of respondents, many of whom did not classify their own research as HSR and therefore deemed their participation unnecessary. Initial high levels of interest by faculty to participate in the assessment waned quickly resulting in FPs having to first determine why participation rates were low. At MUSOPH, Kenya, the FP approached each faculty member in his or her office to better understand the lack of interest. The purpose seemed unclear and length of the tool seemed to be overwhelming, thereby serving as deterrents. In the case of NURSPH, Rwanda, the FP explained that “*once everyone in the institution knew what was happening, they were responsive. But those who weren’t informed from the onset, they thought they weren’t part of the initiative and didn’t take time to respond.*”

Once the reasons for low participation were determined, FPs explored innovative ways to encourage engagement. At KSPH, DRC, the FPs, with support from the Vice Dean of the School of Medicine, invited all senior and junior staff of the SPH to attend a journal club about HSR in advance of the self-assessment. At SPHUoN, Kenya, and MUSOPH, Kenya, the FPs had a sensitization session with department heads and board members during their strategic meetings. Informing staff that the assessment was the first step in a longer process in which capacity development plans were the ultimate aim was key to assuring them that their input was valuable and would lead to change.

#### Resources challenges

Perhaps the most-cited challenges were those regarding time and human resources. Although all teams met in June 2011 and agreed on a protocol, the time it took to receive institutional review board approval as well as contact all necessary key informants within the following 3 months proved overly optimistic. Furthermore, according to FPs at SPHUoN, Kenya, and MUSPHSS, Tanzania, the inclusion of extra team members to assist with the roll out also provided an opportunity to mentor junior faculty and students. Financial support was in the range of $7,000 per school and all SPHs felt that the budget was sufficient for such an exercise.

All schools were able to access digital recorders either by borrowing or buying them for the purposes of the assessment. In cases where key informants refused to be taped, extensive notes were taken to ensure that the necessary information was still captured. Key informants were sometimes located far away, so scheduling and keeping appointments proved challenging at times. As articulated by the MUSPHSS, Tanzania, FP, “*The problem isn’t the number of interviewers; it is the availability of data and people to interview! If people don’t have time, or the data doesn’t exist – there is nothing that can be done, regardless of how many interviewers there are!*” Each team, however, found a strategy that proved effective.

“*When I finally decided to just go to the* [external stakeholder] *offices and ask for 5 minutes of their time, I often received 30 minutes to an hour of discussion because they were interested. So it is negative that they didn’t appear to value academia when solicited by an email or call, but positive when they actually had a chance to engage in dialog.*” (FP, MUSPHSS, Tanzania)

In the case of Kenya and DRC, many external persons completed the HSR priority setting exercise via email rather than through face-to-face interviews due to complex travel schedules or being located elsewhere in the country. These two contexts also permitted the teams to leverage existing networks and opportunities to access key informants as described below:

“*A web forum* [of external stakeholders] *in Congo* [DRC] *exists. Parts of the questions of the interview were sent to the forum and five responses came this way. Others were met individually after phoning them.*” (FP, KSPH, DRC)

“*University of Nairobi is currently working on a curriculum for a Masters in Leadership and Health Services management. There was stakeholders workshop for these discussions and so I took advantage of that and managed all key informant interviews* [for HSR priority setting] *in one day.*” (FP, SPHUoN, Kenya)

#### Data availability

Acquiring all of the necessary information for the institutional profile was difficult. For instance, garnering financial data relevant to grants and research at the university was frustrating due to fragmented accountability structures and aggregated accounting systems.

“*We had to meet with the project office and dig up data. We also sent a form to the principal investigators if they would be able to provide info on each of their research projects but this was difficult.*” (FP, KSPH, DRC)

Documenting research outputs was cumbersome for two reasons. First, HSR was poorly understood and so faculty did not list publications that should be classified as HSR. Second, there were difficulties in locating other types of non-peer reviewed research outputs. In the case of CPHMS, Ethiopia, all research outputs were tabulated so as to ensure wide capture and to avoid missing HSR-relevant outputs. MakSPH, Uganda, had similar challenges:

“*We have a list of dissertations and went through each of them to tease out which are related to HSR. With respect to publications, we had to actually read much of the article to know if it was HSR and fit within the definition agreed upon during the June 2011 Uganda meetings.* [We also] *looked at courses that the faculty coordinate to determine if the individuals are HSR faculty.*” (FP, MakSPH, Uganda)

FPs suggested the need to find a better way of classifying, archiving, and referencing research outputs. Short of creating a resource center, the FP at MUSOPH, Kenya, suggested that screening and analyzing research publications routinely submitted to the Dean’s office every year would be more efficient and reliable than requesting abstract submissions from each faculty researcher, as they did during this assessment. Similarly the FP at SPHUoN, Kenya, expressed that having access to the quarterly updates submitted by faculty to the Director of the SPH could have enhanced capture and documentation of outputs.

### Outcomes of the capacity assessment exercise

In this section, we describe what transpired in each of the SPHs as a consequence of conducting the capacity assessment. Capturing the strengths, weaknesses, challenges, and opportunities of HSR in each SPH served as a useful basis for enhancing institutional strategic plans. As mentioned by the FP at KSPH, DRC, the assessment provided the justification for investment in capacity strengthening in each SPH and culminated in the creation of a capacity development plan for each SPH.

“*The school had no policies or guidelines on human resource development and so these have been recognized. Insufficient persons with qualitative research skills emerged from the assessment as well. Absence of an HSR agenda in the school was highlighted. Also, people realized that we at the SPH do not communicate very well. We do not (seem to) care about media. Knowledge translation need and capacity was low. It was a concept that was new and unrealized so it has become an area that KSPH is now paying more attention to.*” (FP, KSPH, DRC)

The exercise also highlighted that HSR is embedded across multiple classes and courses, rather than having an identity in its own right. This may be problematic for students seeking to build HSR skills as they struggle to identify the right classes to take, as well as potentially undermining a systematic approach to teaching HSR.

“*Methods for HSR are different than Epidemiology and Biostatistics. It would be good to know from beginning about the differences – especially in the dissertations so that the right resources can be used to inform the student research.*” (FP, MakSPH, Uganda)

Although the primary goal of the initiative was to conduct an assessment that would inform each SPH of its individual capacity to conduct HSR, all seven SPHs mentioned additional outcomes. For instance, discussions around the assessment raised the profile of HSR. These provided NURSPH, Rwanda, with a better sense of the range of research studies currently underway and where each falls in the HSR spectrum. MUSPHSS, Tanzania’s FP echoed this point by stressing that definitions of terms were not enough and that awareness raising and discussion were critical to ensuring a common understanding of HSR.

A notable outcome was that the assessment results and the outreach to external stakeholders provided insight about policy-relevant research agendas. Discussions with key informants seem to have increased the profile of some of the SPHs within their own countries. For instance, the lead role that KSPH, DRC, played in convening an HSR priority setting exercise led to renewed interest in involving KSPH, DRC, in healthcare reform in the DRC. Similarly, at CPHMS, Ethiopia, results of the assessment led the university’s Academic Commission to set up a new task force in early 2012 to investigate the reasons behind the low levels of research at the university and propose solutions. Moreover, the FP for the organizational assessment at CPHMS, Ethiopia, was asked to serve on this task force. The task force was subsequently supported by the establishment of a HSR team in September 2012. Exposure to external stakeholders such as colleagues at WHO and at the MOH was mentioned as another important outcome for the CPHMS, Ethiopia, team.

Last but not least, the exercise created a sense of collegiality amongst the seven schools that galvanized collective action for shared interests. For instance CPHMS, Ethiopia, initiated a workshop with JHU on HSR methods training for all partners; MUSOPH, Kenya, took the lead on creating a new course on HSR for all the partners and spearheaded the crafting of grant proposals for funding agencies; and all FPs united in the publication of several manuscripts showcasing the results of the capacity assessments [25-27].

## Discussion

The multidisciplinary nature of HSR inevitably proffers multiple interpretations of what it encompasses. Associated with this nebulous understanding is confusion about the types of individual skills and institutional structures needed to support HSR. While such problems have been recognized previously [4,32], and were accordingly addressed in the original tool, the explanations and examples of HSR provided were not sufficient, resulting in a need to organize additional activities for faculty sensitization to HSR. Similar initiatives should consider raising awareness and providing clarity on time commitments as a first step in such an organizational assessment. Taking into consideration the extent to which HSR was a novel idea to some faculty, it would seem that interviewer-administered self-assessments were important for helping faculty understand the concept, clarifying questions, and enhancing completion rates of the assessments. The depth of responses, therefore, is likely to vary between assessments that were self-administered and those that were interviewer-administered. Similarly, the content of responses during face-to-face interviews is likely to vary significantly from those returned via email. Ultimately, the self-assessments provided faculty perceptions of organizational capacity. The institutional profiles provided more objective data regarding some of the issues that were considered in the self-assessments. Triangulation of sources served to validate the findings in some instances, particularly with objectively verifiable data (e.g., number of publications). However, in other cases (e.g., access to databases) the post-assessment workshops served as a conduit for dialogue to reconcile different perceptions and arrive at shared understandings.

Experiences across the seven schools suggest that it would be prudent to allocate approximately 6 months to a project such as this and target a period in the year when faculty and leadership have time to dedicate to the initiative. With the volume of information that needs to be collected as well as the skills and time needed for the various steps of the assessment, it would be necessary to enlist the assistance of 3 to 5 persons in addition to the FPs, depending on the size of the SPH.

When implementing an assessment across schools or even across departments, the importance of collective creation, consultation, and acceptance of the tools cannot be overemphasized. As was demonstrated in the study described in this paper and elsewhere [19,25-27,33], creating an atmosphere of collaboration and deliberation at the very beginning of the assessment process offered fertile ground for shared learning throughout. Reviewing the assessment tools together and in person prior to implementation allowed for a better understanding of the content, an opportunity to pilot test and refine the questions, and the establishment of a standardized methodology across the seven schools. This uniformity provided a basis for cross-institutional comparison and learning similar to that experienced by Mirzoev et al. when performing HSR capacity assessments across a different set of African SPHs [19]. Furthermore, building in flexibility for adapting materials so that they correspond to the relevant contexts is equally critical in unearthing the reasons why differences across the institutions are observed. Given that all sections of the tool were important for gaining an understanding of the institution as a whole, as well as perceptions of staff, it may be necessary to consider alternative ways of administering the tool so that all sections could be completed more quickly and easily*.* Although access to key external stakeholders led to positive outcomes in several cases, the purpose of setting a research agenda was not wholly fulfilled. Alternative strategies for stakeholder engagement need to be explored, perhaps through more deliberative dialogues [34].

From an analysis of the process of executing HSR institutional capacity assessments across these seven SPHs, it would appear that four factors play a critical role: leadership support, a conceptual understanding of HSR, adequate resources, and data availability.

An ex post facto reflection such as this allows institutions to learn from the experiences of others. However, reliance on FPs as being the sole source of these reflections subjects the interpretations to response bias. Furthermore, the process evaluation occurred four months after the completion of the organizational capacity assessments and therefore may also suffer from recall bias. Given the collaborative nature of the project and the willingness to learn from the experiences of each of the SPHs, we expect that there is little incentive for FPs to skew their responses. Nevertheless, it may have been interesting to conduct interviews with other members of the assessment team, select respondents, and the Dean. Clearly, this paper has not sought to validate the items used in the self-assessment questionnaire or assess their reliability: our focus was principally on understanding the processes and consequences of implementing the capacity assessment tool. The sample size at each participating SPH in our study was too small for any rigorous assessment of validity or reliability, but future studies with larger samples could usefully contribute to this.

## Conclusions

The HSR capacity assessments permitted various aspects of capacity at the SPHs to be explored and analyzed. The intent was to roll out a self-led process for capacity assessment in order to devise HSR capacity development strategies for the SPHs and identify where capacity development investments would have the greatest impact. The assessments led to a number of notable outcomes. First, the exercise galvanized interest in HSR among faculty and leadership across all seven SPHs. Second, the assessment was critical in the development of new capacity development plans for each of the SPHs. Third, it spurred action for implementing joint activities such as course development, organizational activities such as local awareness raising, and individual activities such as training in HSR. In addition, the SPHs are independently and collectively seeking funding for the various aspects of their capacity development plans. Fourth, the assessment stimulated the involvement and interest of key external stakeholders in collaborating with the SPHs. The tool (and exercise overall) has therefore demonstrated utility for generating much more than just an assessment of SPH capacity. It provides data that can be analyzed in a variety of ways [19,25-27] yielding unexpected and valuable side benefits. Furthermore, unlike other institutional capacity assessments that are intended for one particular organization [33] or externally sourced [8], this paper provides insight into an individually endogenous, collectively unified, and environmentally contextualized process across several organizations.

In addition to raising the profile of HSR in each SPH and among that country’s healthcare leadership, an assessment such as this inevitably raises expectations about the next steps. The gaps identified through such a process often require strategic policy and practice changes that are seldom overcome without financial investments. Without the financial and human resources to accompany recommended changes, any momentum gained from galvanizing staff motivation could diminish quickly. This leads one to wonder, what is the best way to plan for capacity development in tandem with resource mobilization with a goal of promoting organizational performance enhancement?

## Abbreviations

CPHMS: Jimma University College of Public Health and Medical Sciences, Ethiopia; CHSRF: Canadian Health Service Research Foundation; FP: Focal person; HEALTH Alliance: Higher Education Alliance for Leadership Through Health; HSR: Health systems research; IDRC: International Development Research Centre, Canada; JHU: Johns Hopkins University, USA; KSPH: Kinshasa School of Public Health, Democratic Republic of Congo; MakSPH: Makerere University College of Health Sciences School of Public Health, Uganda; MUSOPH: Moi University School of Public Health, Kenya; MUSPHSS: Muhimbili School of Public Health and Social Sciences, Tanzania; NURSPH: National University of Rwanda School of Public Health, Rwanda; SPH: School of public health; SPHUoN: University of Nairobi School of Public Health, Kenya.

## Competing interests

The authors declare that they have no competing interests.

## Authors’ contributions

All four authors were closely involved in supporting the implementation of the self-assessment tool. SB designed the first draft of the tool. NJ, DL, and EE conducted the first workshop with representatives from the different SPHs where the tool was adapted to meet local needs (June 2011). NJ revised and finalized the tool upon user feedback. All four authors then participated in the analysis and dissemination workshop (December 2011), and reviewed the individual self-assessment reports. NJ and DL conducted semi-structured interviews with all seven of the lead faculty in the different SPHs to document the implementation process in each country. All authors read and approved the final manuscript.

## Authors’ information

SB is CEO of the Future Health Systems (FHS) research programme consortium. DL and NJ worked with FHS from Johns Hopkins University, and EE leads the Uganda FHS team.

## Supplementary Material

Additional file 1 **Health Systems Research in the HEALTH Alliance Schools of Public Health: capacity development and collaborative research: Health Systems Research Capacity Assessment.** This additional file contains the complete set of assessment tools that were used by the seven schools of public health that are members of the HEALTH Alliance. Click here for file
