# Supplementary material for: Institutional capacity for health systems research in East and Central African schools of public health: experiences with a capacity assessment tool
Source: Health Res Policy Syst. 2014 Jun 2;12:21. doi: 10.1186/1478-4505-12-21 (PMC4067125; doi:10.1186/1478-4505-12-21)
Supplement: Additional file 1 — Health Systems Research in the HEALTH Alliance Schools of Public Health: capacity development and collaborative research: Health Systems Research Capacity Assessment. This additional file contains the complete set of assessment tools that were used by the seven schools of public health that are members of the HEALTH Alliance. [file 1478-4505-12-21-S1.docx]

**Health Systems Research in the HEALTH Alliance Schools of Public Health: capacity development and collaborative research**

**The HEALTH Alliance and the Future Health Systems Consortium**

**Health Systems Research Capacity Assessment**

**Introduction**

At a meeting of the Health Alliance, Deans of the Schools of Public Health in East Africa, which took place in Kigali in February 2011, it was agreed that the Schools of Public Health that make up the HEALTH Alliance, together with the Future Health Systems Consortium would work collaboratively to develop capacity in health systems research in the region.

This note describes the proposed first phase of this work during which capacity assessments and research priority-setting processes will be carried out. It is intended that the first phase of work be implemented during the period February –September 2011.

**Objectives**

- Through a process of self-assessment, develop a clear picture of existing capacities for health systems research at the HEALTH Alliance Schools of Public Health
- Draft and build consensus around health systems research capacity development strategies for each School of Public Health and work plans for implementing these
- Make an initial and rapid assessment of health systems research priorities in the different countries involved in the Health Alliance

**Approach**

We propose that the assessments be **self-assessments** and **Key Informant Interviews of internal and external stakeholders**, led by staff from the institution/SOPH concerned. This approach is proposed because the intention of the assessment exercise is primarily to provide a systematic method for the SOPH to reflect on its strengths and weaknesses with respect to HSR and to stimulate discussion and agreement on what kind of strategies would be most effective to help develop HSR capacity. Note that the assessment tool is designed to assess organizational capacity for HSR, not individual researcher capacity. There are some data from the assessment exercise that we may wish to use as a “baseline” against which changes in capacity can be assessed; this is very much a secondary objective. Further, in addition to the self-assessment we are suggesting that the lead staff from each institution produce an institutional HSR profile, which would be a short document reporting key performance indicators for HSR, and which could be used for baseline indicators of capacity.

Where possible the Focal Persons leading the assessments should be supported by external facilitators who can help facilitate group discussions, advise on the interpretation of questions in the assessment tool, and help the SOPH understand better how it might compare with other organizations. The external facilitators may come from other organizations within the HEALTH Alliance, or from the Future Health Systems team.

Three primary pieces of data collection are proposed:-

- Self-assessment of existing capacity for health systems research. A draft tool and approach for this self-assessment is proposed in COMPONENT A. The tool draws heavily on [1][2][3][4].
- Profile of health systems research within the institution. This is a simple form to collect *objective* data about the number of staff working in health systems research, the skills represented, and the type of work conducted. It complements the self-assessment described above (see COMPONENTS B and C).
- A “quick and dirty” exercise to consult with key stakeholders in-country, and researchers at the School of Public Health, to try to determine priority research questions in the health systems field. It is proposed that a total of 5-8 interviews be done in each country with outside stakeholders. An approach for selecting stakeholders and potential interview questions are proposed in COMPONENT D.

**Implementation process**

We propose the following steps in the application of the assessment tool:

**STEP 1**

Focal Persons for each participating SOPH are identified (one-two people in each case), and these leads in turn take responsibility for identifying ALL individuals (e.g. the deans, deputy deans, head of departments, faculty and other key people) in their institution who have a stake or an interest in HSR. Note that this group is likely to include people at very different levels, from those who are responsible for data collection in the field for HSR studies, or keeping appropriate HSR resources in the library, to more senior university leaders who may not engage in HSR themselves but have a stake in seeing HSR flourish in their organization. It is important to attempt to capture as many people as possible involved in HSR does the self-assessment.

**STEP 2**

Each of the identified individuals is asked to complete, on their own the self assessment questionnaire (COMPONENT A). Consent form can be found in Annex 1.

**STEP 3**

The Focal Persons also collect relevant data to complete a short “HSR” profile questionnaire for the institute which provides basic data on the status of HSR within the organization (COMPONENT B AND C). Consent form can be found in Annex 2.

The Focal Persons arrange for a “quick and dirty” exercise to consult with key stakeholders in-country, and researchers at the School of Public Health, to try to determine priority research questions in the health systems field (COMPONENT D)

**STEP 4**

The Focal Persons compile responses from the self-assessment questionnaire (COMPONENT A), HSR profile (COMPONENTS B and C) as well as the stakeholder assessment (COMPONENT D), so as to present a composite picture of how respondents view organizational capacity in health systems research, so as to produce a short report on priority research questions

**STEP 5**

A first meeting of participants is organized to discuss responses to the self-assessment questionnaire (A proposed agenda for the meeting fill follow shortly).

If a very large group of people have completed the self-assessment questionnaire then it may be necessary to break the meeting up into small groups, but ideally there would be one meeting with everyone participating together.

The objective of the meeting is to share responses, but most importantly to come to agreement about the key capacity strengths and weaknesses, and the underlying reasons for these.

The group may wish to do this by seeking to come to a consensus on appropriate scores for each item measured, or may wish to approach this in a more qualitative fashion. It may be useful to have an external facilitator facilitate this meeting. The HSR organizational profile can be used to support discussions.

**STEP 6**

Based upon this first meeting the Focal Persons (perhaps with support from the external facilitator) write up the key findings from the self-assessment.

Based upon the self-assessment findings, a draft outline of a capacity development strategy is developed by the Focal Persons and the external facilitator.

**STEP 6**

A second meeting with participants is organized with the aim of agreeing a package of strategies that would be effective at building upon organizational strengths in HSR and addressing organizational weaknesses (an agenda for this meeting will also follow shortly). Based on this discussion the organizational capacity development strategy is further refined.

**STEP 7**

A workshop is held that brings together all the different Schools of Public Health in the HEALTH Alliance (probably in November 2011). Each team presents their own capacity assessment, and the capacity development strategy and research priorities. A collective plan is developed that will allow some activities that will support capacity development across all of the Schools (e.g. workshops for curriculum development in health systems research), as well as the individually tailored plans.

The Future Health Systems Project will support some of the capacity development activities, additional sources of support may be needed for other elements of the plan.

**Products**

1. Reports on existing capacity for Health Systems Research at each of the institutions that make up the HEALTH Alliance
2. Capacity development plans for each of the institutions within the HEALTH Alliance
3. Short report on priority health systems research questions in each of the HEALTH Alliance countries
4. Future Health Systems/HEALTH Alliance work plan that describes how the two groups will work together to strengthen health systems research capacity and undertake collaborative health systems research over the coming years.
5. Proposal to other funders to support health systems research through the HEALTH Alliance

**Figure showing process for development of capacity assessments and workplans**

**COMPONENT A: SELF ASSESSMENT**

*Dear esteemed colleague,*

*Thank you for your participation in this institutional assessment. Your impressions, perceptions, thoughts and feedback are extremely important and will feed into capacity development initiatives at our School of Public Health.*

*This is the first step in the assessment of health systems research capacity at the different Schools of Public Health that constitute the Health Alliance. Everyone who has any interest in health systems research at our School of Public Health is being asked to complete this short questionnaire. We will then compile all of your responses and organize a meeting where we can discuss the conclusions emerging from this exercise. Please provide us with your honest and frank assessment of current strengths and weaknesses at the School as this assessment will feed into the development of a capacity development strategy.*

*There is sometimes a lack of clarity about what constitutes health systems research. Health systems research has been defined in this context as follows:*

**“Health Systems Research is interdisciplinary in nature and aims to provide policy and practice relevant information, so as to improve the health system as a whole by addressing the goals of equity, efficiency, effectiveness, and sustainability - ultimately leading to improved health status”**

*The results of these self-assessments will be one of several sources of data upon which a capacity development plan will be constructed. All faculty who respond to this survey will be invited to discuss the aggregate results and contribute to discussions on what needs prioritizing with respect to strategies for institutional capacity enhancement. Discussion will also encourage ideas for how such capacity development initiatives could be operationalized.*

*Please note that your responses are anonymous and your feedback will remain confidential. Answers will be aggregated with those of others so as to minimize any possibilities of attribution.*

*The survey should take approximately XXXX minutes of your time. When complete, kindly return the hard copy to XXXX(insert name of focal person) or return it via email to XXXX*

*We request that you return this survey to the above mentioned contact person no later than*

*XXXXXX (Kindly insert date)*

*Sincerely,*

*NAME of Dean or focal person.*

Here are some examples of research considered to be “health systems research”:

- Vouchers to address barriers to accessing maternity care in Uganda: researchers conducted formative research with local communities to identify barriers to the use of skilled birth attendants. The cost of travelling to health care facilities was determined to be a major barrier. The researchers designed, helped to implement and evaluated a scheme that provided vouchers to pregnant women that enabled them to pay for local forms of transport to get to the nearest health care facility at the time of their delivery.(Ekirapa-Kiracho et al 2011)
- In South Africa researchers studied the position of different stakeholders regarding a proposed health insurance reform. They identified which actors supported the reform and which opposed it and the relative power of different actors. Based on this they developed a proposed strategy for managing the interests of the different actors to allow the reform to move forward. (Thomas and Gilson 2004)
- A multi-country study conducted in Tanzania, Bangladesh, Peru and other sites, evaluated the impact of the introduction of the integrated management of childhood illness program and sought to establish the causal pathways through which impacts occurred. The researchers observed that frequently the programme was not fully implemented and as a consequence the impact of the program on child health outcomes was less than anticipated (Bryce et al 2005)
- In Kenya an ethnographic study sought to understand how community based distributors in family planning programs sought to use their jobs as a means to achieve their own personal goals. The study demonstrated how the distributors’ concerns to build their own respect within the community and to avoid blame for any possible negative outcomes, affected the type of services that they were willing to provide (Kaler and Cotts 2001)
- In India, a cluster randomized trial was used to assess whether or not alternative design features in a community health insurance scheme increased the equity of the scheme or not (Ranson et al 1007)

Here are some real examples of studies that we do NOT consider to be health systems research:-

- In Tanzania researchers examined the cost-effectiveness of iron supplementation and malaria chemoprophylaxis in the prevention of anaemia and malaria among infants (Gonzalez et al 2000)
- In Kenya researchers looked at the causes and outcome of young infant admissions to a district hospital (English et al 2003).
- Lab researchers identified a draft sequence of the genome of *Aedesaegypti*, the primary vector for yellow fever and dengue fever,(Nene et al 2007)

**Glossary of terms:**

***Health system***: “is the sum total of all the organizations , institutions and re-sources whose primary purpose is to improve health. These include formal health services; action by traditional healers; all use of medication whether pre-scribed by a provider or not; home care for the sick; traditional public health activities such as health promotion and disease prevention and health enhancing interventions including road and environmental safety improvements.” (World Health Report 2000: Health Systems- Improving Performance)

***Health Systems Research*:** HSR is interdisciplinary and aims to provide policy and practice relevant information, which will improve the health system as a whole, by addressing the goals of equity, efficiency, effectiveness, and sustainability, which ultimately leads to, improved health status (HEALTH Alliance, Africa hub meeting June 2011)

***Research Use and Uptake:*** *(Also referred to as Knowledge Translation and Exchange, research communication etc)* Knowledge Translation is the synthesis, exchange, and application of knowledge by relevant stakeholders to accelerate the benefits of global and local innovation in strengthening health systems and improving people’s health. (WHO, 2005). The intent is to encourage utilization of research results by tailoring the packaging of results for the intended audience and leveraging different mechanisms to reach them.

***Policy maker****:* A person with power to influence or determine policies and practices at an international, national, regional, or local level (Minister, permanent secretary, Board of Management, Board of directors, Chancellor etc…)

***Decision-maker:*** Someone plays a key role in the administration (and leadership) of an organization (eg chancellor, director, manager, academic administrator, District medical officer etc…)

***Research Outputs:*** Activities and products resulting from research pursuits and results (eg articles in peer reviewed publications, research summaries, policy briefs, leaflets/brochures, policy dialogues, public forums, radio spots, videos, theatre, talkshows etc)

***Capacity strengthening:*** Ongoing process of empowering individuals, institutions, organizations and nations to: 1) define and prioritize problems systematically; 2) develop and scientifically evaluate appropriate solutions and 3) share and apply the knowledge generated (Lusthaus et al, IDRC 1995)

***Capacity Development (CD):***The process by which individuals, groups and organizations, institutions and societies/countries develop, enhance and organize their systems, resources and knowledge; all reflected in their abilities, individually and collectively, to perform functions, solve problems and achieve objectives; to understand and deal with their development need in a broader context and in a sustainable manner (UNDP, 1997)

**SECTION ONE: INSTITUTE PARTICULARS AND ORGANISATION**

*You are kindly requested to provide all the information required below*

**A.1.1**Department: _____________________________________________

**A.1.2**Job Title: _________________________________________________________

**A.1.3**Please fill in the qualifications table below? (Put a tick next to all that apply)

| **Disciplinary focus of training** | **PhD** | **Masters** | **Bachelors** | **Diploma** |
| --- | --- | --- | --- | --- |
| **Economics** |  |  |  |  |
| **Social Science (anthropology, sociology)** |  |  |  |  |
| **Political science/policy analysis** |  |  |  |  |
| **Epidemiology** |  |  |  |  |
| **Clinical** |  |  |  |  |
| **Biostatistics** |  |  |  |  |
| **Health services research** |  |  |  |  |
| **Public health Subdisciplineeg: MCH, Int’l health (Please specify)** | (Please specify) | (Please specify) | (Please specify) | (Please specify) |
| **Other (Please specify)** | (Please specify) | (Please specify) | (Please specify) | (Please specify) |

**A.**1.4 Academic position

____Professor

____Associate professor

_____Senior Lecturer

_____Junior lecturer

___Dean

____Vice Chancellor

____Chancellor

_____Principal

____Other (please specify)__________________

**A.1.5** For how many years have you worked at this School? ___________

**A.**1.6 how long have you been collaborating with the SOPH (if not a part of it)?

**A.**1.7 Have you conducted HSR, engaged in HSR, or have an interest in HSR? (if no, stop here and please return this to the focal person)

A.1.8 Gender ___M ___F

A.1.9 Age ________yrs

**SECTION TWO: CAPACITY DEVELOPMENT AND COLLABORATIVE RESEARCH IN HEALTH SYSTEMS**

*As a way of assessing to what extent your institution can carry out capacity development and collaborative research in Health Systems Research, you are kindly requested to answer the following questions. The areas to be covered are divided into dimensions, each with statements that require responses on a five-option likert scale.*

Please put a tick in the box that corresponds most closely to how much you agree with each statement. There is no right or wrong answer. We are interested in your opinion.

|  | 1  Strongly disagree | 2  Disagree | 3  Neither agree nor disagree | 4  Agree | 5  Strongly agree |
| --- | --- | --- | --- | --- | --- |
| ***Forces in the external environment*** | | | | | |
| 1. Key institutions in this country, such at the Ministry of Health, have a strong interest in health systems |  |  |  |  |  |
| 1. There are adequate possible funding opportunities for health systems research at the moment |  |  |  |  |  |
| 1. There are adequate organizations in this country which are interested in using health systems research findings |  |  |  |  |  |
| ***Organizational motivation and commitment*** | | | | | |
| 1. Our School of Public Health (SOPH) places a high priority on the conduct of original research |  |  |  |  |  |
| 1. Our SOPH places a high priority on health systems research |  |  |  |  |  |
| *Intentionally left blank* | --- | --- | --- | --- | --- |
|  | 1  Strongly disagree | 2  Disagree | 3  Neither agree nor disagree | 4  Agree | 5  Strongly agree |
| ***Organizational capacity*** | | | | | |
| 1. I feel confident that there are individuals in this SOPH who can provide high level leadership for HSR |  |  |  |  |  |
| 1. This SOPH has adequate number of individuals with strong quantitative research skills who are interested in applying them to HSR |  |  |  |  |  |
| 1. This SOPH has adequate number of individuals with strong qualitative research skills who are interested in applying them to HSR |  |  |  |  |  |
| 1. There are an adequate number of researchers in this SOPH who are interested in HSR. |  |  |  |  |  |
| 1. There are adequate library resources in this SOPH to support research in health systems |  |  |  |  |  |
| 1. We have good access to the kind of databases necessary to conduct HSR in this SOPH |  |  |  |  |  |
| 1. We have access to datasets necessary to conduct HSRhere |  |  |  |  |  |
| 1. This SOPH offers courses relevant to HSR |  |  |  |  |  |
| 1. Courses provided on HSR draw upon a wide body of appropriate literature and teaching materials |  |  |  |  |  |
| 1. Faculty in this SOPH have adequate knowledge to teach HSR |  |  |  |  |  |
|  | 1  Strongly disagree | 2  Disagree | 3  Neither agree nor disagree | 4  Agree | 5  Strongly agree |
| 1. There are many *undergraduate* students at this SOPH who are interested in Health Systems Research |  |  |  |  |  |
| 1. There are many *graduate* students at this SOPH who are interested in HSR |  |  |  |  |  |
| 1. There is flexible funding from the government budget, not tied to any specific project, which is available at this SOPH to support Health Systems Research |  |  |  |  |  |
| 1. This SOPH is able to produce high quality proposals that lead to funded grants for Health Systems Research |  |  |  |  |  |
| 1. Researchers at this SOPH have the time to draft academic papers based upon Health Systems Researchthat they conduct |  |  |  |  |  |
| 1. Researchers at this SOPH have the motivation to write publishable papers based upon Health Systems Researchthat they conduct |  |  |  |  |  |
| 1. Researchers at this SOPH have the skills to write publishable papers based upon Health Systems Researchthat they conduct |  |  |  |  |  |
| 1. Researchers at this SOPH have the time to disseminate their findings to policy makers through knowledge translation mechanisms such as policy briefs or policy dialogues or one-on-one discussions etc. |  |  |  |  |  |
|  | 1  Strongly disagree | 2  Disagree | 3  Neither agree nor disagree | 4  Agree | 5  Strongly agree |
| 1. Researchers at this SOPH have the motivation to disseminate their findings to policy makers through knowledge translation mechanisms such as policy briefs or policy dialogues or one-on-one discussions etc. |  |  |  |  |  |
| 1. Researchers at this SOPH have the skills to disseminate their findings to policy makers through knowledge translation mechanisms such aspolicy briefs or policy dialogues or one-on-one discussions. |  |  |  |  |  |
| 1. This SOPH has a strong communications staff and capacity to effectively communicate HSR findings to *many* different audiences |  |  |  |  |  |
| ***Research support capacity:*** This SOPH has capacities in the areas of: | | | | | |
| 1. development and writing of research proposals (technical and scientific support) |  |  |  |  |  |
| 1. development and writing of research proposals (admin support) |  |  |  |  |  |
| 1. budgeting and financial management, |  |  |  |  |  |
| 1. publication of research in peer reviewed journals |  |  |  |  |  |
| 1. production of a variety of different outputs (knowledge translation) and research uptake |  |  |  |  |  |
|  | 1  Strongly disagree | 2  Disagree | 3  Neither agree nor disagree | 4  Agree | 5  Strongly agree |
| 1. Individual interaction and communication with decision makers/policy makers? |  |  |  |  |  |
| ***Inter-institutional linkages*** | | | | | |
| 1. This SOPH has strong institutional links to the MOH/units within the MOH with an interest in HSR |  |  |  |  |  |
| 1. Ministry of health officials value the evidence that we provide and draw upon it in their work |  |  |  |  |  |
| 1. This SOPH has strong institutional linkages to health facilities and health staff who can be engaged in HSR |  |  |  |  |  |
| 1. Health facilities and health staff value the evidence that we provide and draw upon it in their work |  |  |  |  |  |
| 1. This SOPH has strong institutional linkages to other organizations engaged in HSR in our country. |  |  |  |  |  |
| 1. This SOPH has strong institutional linkages to other organizations engaged in HSR in other countries |  |  |  |  |  |
| 1. This SOPH has strong institutional linkages to NGOs in our country which are interested in health systems |  |  |  |  |  |
| 1. This SOPH has strong institutional linkages to media organizations. |  |  |  |  |  |

*End of Survey. Thank you for your time. I look forward to seeing you at the discussion forum.***COMPONENT B: ORGANIZATIONAL HSR PROFILE – I**

*ATTN: focal persons*

*This part of the institutional assessment seeks to capture the overarching governance mechanisms, strategies and current capacities in the SOPH. There are four sections to COMPONENT B. Objective and comprehensive capture will allow for more in-depth discussions among faculty during the faculty forums that will seek to identify the gaps in capacity as well as provide recommendations and strategies for prioritizing as well as filling those gaps. The discussion forum will depend heavily on the information that is gathered through this organizational HSR profile as well as the self-assessments in COMPONENT A.*

*Kindly fill this template out* ***electronically*** *due to the ability to expand the cells to fit all relevant information. It may also be easier during the process of data entry and analysis.*

**Glossary of terms:**

***Health system***: “is the sum total of all the organizations , institutions and re-sources whose primary purpose is to improve health. These include formal health services; action by traditional healers; all use of medication whether pre-scribed by a provider or not; home care for the sick; traditional public health activities such as health promotion and disease prevention and health enhancing interventions including road and environmental safety improvements.” (World Health Report 2000: Health Systems- Improving Performance )

***Health Systems Research*:**HSR is interdisciplinary and aims to provide policy and practice relevant information, which will improve the health system as a whole, by addressing the goals of equity, efficiency, effectiveness, and sustainability, which ultimately leads to, improved health status (HEALTH Alliance, Africa hub meeting June 2011)

***Research Use and Uptake:*** *(Also referred to as Knowledge Translation and Exchange, research communication etc)* Knowledge Translation is the synthesis, exchange, and application of knowledge by relevant stakeholders to accelerate the benefits of global and local innovation in strengthening health systems and improving people’s health. (WHO, 2005). The intent is to encourage utilization of research results by tailoring the packaging of results for the intended audience and leveraging different mechanisms to reach them.

***Policy maker****:*A person with power to influence or determine policies and practices at an international, national, regional, or local level (Minister, permanent secretary, Board of Management, Board of directors, Chancellor etc…)

***Decision-maker:***someone plays a key role in the administration (and leadership) of an organization (eg chancellor, director, manager, academic administrator, District medical officer etc…)

***Research Outputs:*** activities and products resulting from research pursuits and results (eg articles in peer reviewed publications, research summaries, policy briefs, leaflets/brochures, policy dialogues, public forums, radio spots, videos, theatre, talkshowsetc)

***Capacity strengthening:***Ongoing process of empowering individuals, institutions, organizations and nations to: 1) define and prioritize problems systematically; 2) develop and scientifically evaluate appropriate solutions and 3) share and apply the knowledge generated (Lusthaus et al, IDRC 1995)

***Capacity Development (CD):***The process by which individuals, groups and organizations, institutions and societies/countries develop, enhance and organize their systems, resources and knowledge; all reflected in their abilities, individually and collectively, to perform functions, solve problems and achieve objectives; to understand and deal with their development need in a broader context and in a sustainable manner (UNDP, 1997)

**SECTION ONE:**

**B.1.1**Please attach an organogram of your research unit/SOPH, and the relations between your unit/institute with MOH, national policy makers, national and international research partners and international development partners. *(Note: One organogram may be attached of the institution)*

**B.1.2** Concerning ethical approval of research proposals, where is your IRB/REC located? SOPH_______ University_______National_________

**B.1.2.1**How often does the committee sit? __________

**B.1.2.2**On average how long (in months) does it take for ethical approval of research proposals by the Ethical Committee?**_____________**_mths

**B.1.3***Please append a list of health systems research products for each of the past 5 years. Include within this list all sorts of products from peer review journal articles, to briefing notes, reports to different agencies and media or press releases. How many of each of the following products has there been by year? (N.B: This part may be difficult to fill in but kindly try as much as possible to give all the information)*

*Where possible, kindly include the web/URL link (eg if media report is available online, provide the link to it)*

*Note: In the table below give aggregate numbers but* ***append a list with the details***

|  | 2006 | 2007 | 2008 | 2009 | 2010 | 2011 |
| --- | --- | --- | --- | --- | --- | --- |
| **B.1.3.1** Peer review articles in international journals |  |  |  |  |  |  |
| **B.1.3.2** Peer review articles in national or local journals |  |  |  |  |  |  |
| **B.1.3.3** Briefing notes (Policy briefs) |  |  |  |  |  |  |
| **B.1.3.4** Reports to agencies (MoH, donors, etc) |  |  |  |  |  |  |
| **B.1.3.5** Press releases/media briefings*(incl which type of media – radio/print etc as well as which SOPH member was involved)* |  |  |  |  |  |  |
| **B.1.3.6** Multi-media products (e.g. videos, blogs, radio spots, podcasts, apps) |  |  |  |  |  |  |
| **B.1.3.7**Undergraduate reports |  |  |  |  |  |  |
| **B.1.3.8** Masters theses |  |  |  |  |  |  |
| **B.1.3.9**Doctoral dissertations |  |  |  |  |  |  |

**B.1.4** Is there a designated individual responsible for policy influence and research communication/uptake?____Y ____N

**B.1.4.1**If yes, what is their role, and where are they located in the system?___________________________________________________________________________________________________________________________________________________________________________________________________________________________________

*End of section ONE*

**SECTION TWO: HSR GRANTS/FUNDING CONTRACTSAND GENERAL FINANCIAL RESOURCES**

**B.2Financial Resources:**

**B.2.1**Resource mobilization: You are kindly requested to provide information on the types of funding that your SOPH has received over the past 5 years by filling in the table below*(NOT restricted to HSR).*

*(Potential source of information – grants management office, finance and budget staff, faculty)*

| Type of funding | Source profile  (please tick) | | | Proportion  (please tick) | | | |
| --- | --- | --- | --- | --- | --- | --- | --- |
|  | Gov’t | Local | Int’l | Research | Staff dev | ICT | maintenance |
| **B.2.1.1**Core grant (ie general budget support that is not tied to any specific project) |  |  |  |  |  |  |  |
| **B.2.1.2** Research grant |  |  |  |  |  |  |  |
| **B.2.1.3** Training grants |  |  |  |  |  |  |  |
| **B.2.1.4** Consulting fees |  |  |  |  |  |  |  |
| **B.2.1.5** Training fees |  |  |  |  |  |  |  |
| **B.2.1.6** Government subsidies |  |  |  |  |  |  |  |
| **B.2.1.7** Tuition fees |  |  |  |  |  |  |  |
| **B.2.1.8** Other (please specify) |  |  |  |  |  |  |  |

**B.2.2**SoPH Resource security: Please provide annual data on SOPH revenues for the past FIVE years.

*(Potential source of information – Dean, finance and grants budgeting office, faculty)*

**B.2.3** How many **health systems research grants** and contracts has your SOPH received during the past five years (ie since January 2006)? What was the magnitude of these grants and what was their focus?

*Restricted to HSR - Based on the HSR definition in the glossary provided in this tool, include only grants that partially or fully incorporate HSR.*

| Grant name | Donor/funder | Amount | Period (from – to) *(please provide months if grant or contract was for < 1year)* | Primary focus | Comment *(please note if only a proportion of the grant was for HSR)* |
| --- | --- | --- | --- | --- | --- |
|  |  |  |  |  |  |
|  |  |  |  |  |  |
|  |  |  |  |  |  |
|  |  |  |  |  |  |
| Please add extra rows as necessary | | | | | |

*End of Section Two.*

**SECTION THREE: HSR STAFF**

**B.3.1**How many research staff are there at your SOPH who are **actively** engaged in health systems research? *Please complete the following table indicating the number of research staff by their primary area of focus and the type of degree they possess. (To be filled in by the person collecting data)* While it would be preferable to try to establish what percent of their time each PT or FT faculty spends on HSR, this may not always be easy to determine. However, if possible to collect, kindly include the necessary information.

*(Potential sources of information: faculty, human resources department, staff CVs)*

*(Please note: FT = Full Time; PT = Part Time as per their employment status at the SOPH.)*

| **Disciplinary focus of training** | **PhD** | | | | **Masters** | | | | **Bachelors** | | | | **Diploma** | | | |
| --- | --- | --- | --- | --- | --- | --- | --- | --- | --- | --- | --- | --- | --- | --- | --- | --- |
|  | **Male** | | **Female** | | **Male** | | **Female** | | **Male** | | **Female** | | **Male** | | **Female** | |
|  | **FT** | **PT** | **FT** | **PT** | **FT** | **PT** | **FT** | **PT** | **FT** | **PT** | **FT** | **PT** | **FT** | **PT** | **FT** | **PT** |
| **Economics** |  |  |  |  |  |  |  |  |  |  |  |  |  |  |  |  |
| **Social Science (anthropology, sociology)** |  |  |  |  |  |  |  |  |  |  |  |  |  |  |  |  |
| **Political science/policy analysis** |  |  |  |  |  |  |  |  |  |  |  |  |  |  |  |  |
| **Epidemiology** |  |  |  |  |  |  |  |  |  |  |  |  |  |  |  |  |
| **Clinical** |  |  |  |  |  |  |  |  |  |  |  |  |  |  |  |  |
| **Biostatistics** |  |  |  |  |  |  |  |  |  |  |  |  |  |  |  |  |
| **Health services research** |  |  |  |  |  |  |  |  |  |  |  |  |  |  |  |  |
| **Public health Sub discipline e.g: MCH, Int’l health (Please specify)** |  |  |  |  |  |  |  |  |  |  |  |  |  |  |  |  |
| **Other (Please specify)** |  |  |  |  |  |  |  |  |  |  |  |  |  |  |  |  |
| **Other (Please specify)** |  |  |  |  |  |  |  |  |  |  |  |  |  |  |  |  |
| **Other (Please specify)** |  |  |  |  |  |  |  |  |  |  |  |  |  |  |  |  |
| **Other (Please specify)** |  |  |  |  |  |  |  |  |  |  |  |  |  |  |  |  |
| **Other (Please specify)** |  |  |  |  |  |  |  |  |  |  |  |  |  |  |  |  |
| **Other (Please specify)** |  |  |  |  |  |  |  |  |  |  |  |  |  |  |  |  |

*End of Section Three*

**SECTION FOUR: SCOPE OF HSR TEACHING**

**B.4.1**Which courses does your SOPH teach in HSR?

*Please complete the table below indicating the focus of the course, and the type of HSR topics covered.*

| **Name of course** | **Faculty teaching the course (names)** | **Topics relevant to HSR covered by the course** |
| --- | --- | --- |
|  |  |  |
|  |  |  |
|  |  |  |
|  |  |  |
|  |  |  |
|  |  |  |
|  |  |  |
|  |  |  |
| Please add extra rows if need be. | | |

*End of Section Four*

*End of Component B.***COMPONENT C:ORGANIZATIONAL PROFILE-IIAND KEY COLLABORATORS**

*ATTN: focal persons*

*This part of the institutional assessment seeks to capture the second part of the overarching organizational profile and therefore need not be HSR specific. . There are two sections to COMPONENT C.*

*Objective and comprehensive capture will allow for more in-depth discussions among faculty during the faculty forums that will seek to identify the gaps in capacity as well as provide recommendations and strategies for prioritizing and filling those gaps. The discussion forum will depend heavily on the information that is gathered through this organizational HSR profile as well as the self-assessments in COMPONENT A and part I of the organizational profile captured in COMPONENT B.*

*This section can be done using key informant interviews with senior management at the institution. Note that questions may require extensive probing and strong qualitative interviewing skills.*

*Kindly fill this template out* ***electronically*** *due to the ability to expand the cells to fit all relevant information. It may also be easier during the process of data entry and analysis.*

***Kindly request consent and tape record all interviews for integrity, transcription and reference purposes. Consent forms can be found in Annex 2***

**SECTION ONE:**

**C.1.1** If your SOPH is within a wider organisation, please describe the nature of the link between your group and the wider organisation. For example, who makes decisions on HR issues, funding and direction of work?

*The matrix below will help you to collect additional information about your SOPH. There are six dimensions, please describe the current status with adequate detail, assess the challenges and highlight the assessment of your needs for capacity strengthening. Where relevant, identify where the links between your group and the wider organisation have influence on these issues.*

Date of interview:___________________________________________

|  | **Description of current status** | **Challenges and needs for capacity strengthening** |
| --- | --- | --- |
| *To be filled in by the focal point person (Some consultation may be necessary)* | | |
| **C.1.2Guiding documents and principles** | | |
| **C.1.2.1**Does the organization have a *visible* vision and mission?  *(if so, kindly note where it can be found. URL/web link if on web)* |  |  |
| **C.1.2.2** Is there a current strategic plan?  *(if so, kindly note where it can be found.? Dean’s office? library? URL if on web)* |  |  |
| **C.1.2.3** What process(es) are or were pursued during the creation of the strategic plan?  (eg: probe into participation, key actors, external influences, the length of time etc) |  |  |
| **C.1.2.4**Does the organization have guiding policies for:  *(Note to focal persons: elicit the implementation of policies in your probes)* | Leave this cell blank | Leave this cell blank |
| **C.1.2.4.1**Staff roles and responsibilities |  |  |
| **C.1.2.4.2** Human resources (eg Remuneration (incl internal as well as project related pay), performance Evaluation etc) |  |  |
| **C.1.2.4.3** Resource mobilization |  |  |
| **C.1.2.4.4** Teaching |  |  |
| **C.1.2.4.5** Research |  |  |
| **C.1.2.4.6** Project management |  |  |
| **C.1.2.4.7** Partnerships (MOUs, remuneration, retainer policies, etc) |  |  |
| **C.1.2.4.8** Internal communication |  |  |
| **C.1.2.4.9** External communication |  |  |
| **C.1.2.4.10** publication |  |  |
| **C.1.2.4.11** Capacity strengthening (eg Professional development options, workshops funds, other?) |  |  |
| **C.1.2.5** Which of these policies are available to staff? |  |  |
| **C.1.2.6** Which of these policies are in the public domain? |  |  |
| **C.1.3 Organization structure and governance** | | |
| **C.1.3.1** what is the governance structure? |  |  |
| **C.1.3.2** What is the School Board membership? |  |  |
| **C.1.3.3** How frequently does the School Board meet? |  |  |
| **C.1.3.4** What are the functions of the School Board? |  |  |
| **C.1.3.5 What role does it play in terms of:** | Leave this cell blank | Leave this cell blank |
| **C.1.3.5.1**setting strategic direction |  |  |
| **C.1.3.5.2** influencing which specific activities are pursued, |  |  |
| **C.1.3.5.3** ensuring quality? |  |  |
| **C.1.3.5.4** ensuring financial security for the organization? |  |  |
| **C.1.3.5.5** ensuring mechanisms are in place for accountability and transparency of SOPH activities and processes? (ex: repercussions for academic misconduct, misuse of funds, poor performance of staffetc) |  |  |
| **C.1.3.6** What are the functions of SOPH senior management? (ex: Dean, other boards, commission, management teams etc..) |  |  |
| **C.1.3.7 What role does it play in terms of….:**  NB: if the answer is no to any of the below, kindly include who else may be making the decisions. | Leave this cell blank | Leave this cell blank |
| **C.1.3.7.1 ….**setting strategic direction |  |  |
| **C.1.3.7.2 ….**influencing which specific activities are pursued? |  |  |
| **C.1.3.7.3 ….**ensuring quality of academic processes? |  |  |
| **C.1.3.7.4** ensuring quality of administrative and finance processes? |  |  |
| **C.1.3.7.5** communicating information across the SOPH? |  |  |
| **C.1.3.7.6** appraising and assessing performance of faculty/staff? |  |  |
| **C.1.3.7.7** public relations/relationships? |  |  |
| **C.1.4 Retention and turnover profiles** | | |
| **C.1.4.1** Specify if there are different strategies for recruitment of junior and senior staff. |  |  |
| **C.1.4.2** How easy is it to recruit qualified staff? |  |  |
| **C.1.4.3** What factors have been found successful in attracting qualified staff? |  |  |
| **C.1.4.4** How easy is it to retain qualified staff? |  |  |
| **C.1.4.5** What factors have been found successful in retaining qualified staff? |  |  |
| **C.1.4.6** To what extent are performance appraisals being used to inform retention policies/strategies? |  |  |
| **C.1.4.7**What is the turnover rate in the past three years? |  |  |
| **C.1.4.8** Where have staff leaving SOPH gone to? |  |  |
| **C.1.4.9** What are the main reasons for staff exit? |  |  |
| **C.1.4.10** Are staff morale and satisfaction surveys being conducted? |  |  |
| **C.1.4.11** To what extent do assessments of staff morale and staff satisfaction being used to influence retention strategies? |  |  |
| **C.1.5 Influencing health policies** | | |
| *Enabling environments for evidence based decision making and culture of using evidence among policy makers in their decision making* | | |
| **C.1.5.1** How does the institute ensure policy relevance of its research? (ex: are research topics based on national health priorities? Etc.) |  |  |
| **C.1.5.2 Describe the collaboration mechanisms with the following:**  (Probe whether formal or informal: eg - official requests to attend meetings, etc) | Leave this cell blank | Leave this cell blank |
| **C.1.5.2 .1** policy makers |  |  |
| **C.1.5.2.2** international partners |  |  |
| **C.1.5.2.3** civil society organizations? |  |  |
| **C.1.6 Capacity development and external support** *(Dean to be interviewed)* | | |
| **C.1.6.1** Is there a staff capacity development plan?  (if no, skip to **C.1.6.3)** | Yes _______ No_______  Explain: |  |
| **C.1.6.2** If yes what does it cover? |  |  |
| **C.1.6.3** What are the main capacity development needs for the SOPH now? |  |  |
| **C.1.6.4** What proportion of SOPH spending goes to staff capacity strengthening?  (if possible, please include actual amounts in the numerator and denominators) |  |  |
| **C.1.6.5** What proportion of staff have benefited from capacity strengthening initiatives?  (refer to examples in the glossary re: capacity strengthening) |  |  |
| **C.1.6.6** What capacity development support has the SOPH received from else where? (financial, technical) |  |  |
| **C.1.6.7** What form has this taken [mentoring, external technical assistance, international positions withinthe SOPH]? |  |  |
| **C.1.6.8** Is the SOPH part of any broader capacity development network within the country or internationally? (specify) |  |  |
| **C.1.6.9** Has the SOPH helped to build the capacity for policy makers**?**  If yes, how was this done, who were the facilitators and users?  *(refer to examples in the glossary re: capacity strengthening)* | Yes ________ No______ |  |

*End of Section One*

**SECTION TWO: COLLABORATORS**

**C.2.1** Which are the key organizations **within** your country that your SOPH collaborates with on HSR? *Please list the organizations below, and briefly describe the nature of the collaboration, any challenges and needs for capacity strengthening using the table below*.

| **Name of organization inside your country** | **Brief description of nature of collaboration** | **Challenges and needs for collaboration** |
| --- | --- | --- |
|  |  |  |
|  |  |  |
|  |  |  |
|  |  |  |
|  |  |  |
|  |  |  |

**C.2.2**Which are the key organizations **outside** your country that your SOPH collaborates with on HSR? *Please list the organizations below and briefly describe the nature of the collaboration, any challenges and needs for capacity strengthening using the table below.*

| **Name of organization outside your country** | **Brief description of nature of collaboration** | **Challenges and needs for collaboration** |
| --- | --- | --- |
|  |  |  |
|  |  |  |
|  |  |  |
|  |  |  |
|  |  |  |
|  |  |  |

**C.2.3**Please note how long your SOPH has been in existence, your mission, mandates and the main lines of your current work, considering the areas of work and the balance (in terms of efforts given) among research, policy engagements, broader advocacy, teaching and any other area of activity you conduct.

**C.2.3.1** Has this balance of work changed much over time – and if so, in what way?

*End of Section Two*

*End of Component C***COMPONENT D: IDENTIFICATION OF HEALTH SYSTEMS RESEARCH PRIORITIES**

| Instructions to country teams  *Please identify 5-8 influential policy and decision makers in your country. We suggest that you seek to identify individuals who can represent different types of stakeholder perspectives. For example you may wish to interview*   - Ministry of Health – senior policy maker e.g. Director of Health Services, Health planner or health policy advisor, programme managers for key programmes such as MCH. - Hospital Directors/Medical Superintendents - Representative from medical councils - Representatives from international agencies (e.g. WHO, CDC, UN, UNRO, Ministry of finance) - Provincial and/or district level health official - Senior official in church medical association (If relevant) - Donor representative – if you are able to contact someone at UK DFID then this would be fantastic (as they fund the FHS project) but other donors would be fine. - Representative of local NGO working in the health sector - Parliamentarian - (chair of health committee)   *Below, you will find some initial questions to help you focus the discussion. The questions start with quite broad inquiry and become progressively narrower.*  *Please****record*** *the conversation, and also take detailed notes so that you can develop a synthesis of your discussions later.It may be necessary to involve some help in capturing the discussions.* |
| --- |

DATE of discussion:___________________

Questions

1. What do you think are the main health systems challenges that our country is facing at the moment?
2. What types of policy reforms or new programs is the government considering adopting in order to address these challenges?
3. What are some of the Key health sector reforms in particular health financing, thathave beenoccurring in the country in the last five years to date?
4. What are some of the Key health sector reforms in particular human resourcesthat have been occurring in the country in the last five years to date?
5. What are some of the Key health sector reforms in particular in health systems governance that have beenoccurring in the country in the last five years to date?
6. Can you think of any needed research or evidence that would help you to formulate policies and decisions on these issues?
7. What do you think are some of the HSR priority areas in the country?

*Please note that many policymakers will face lots of difficulties in turning their current policy concerns into research questions and you may need to probe. For example, if the policymaker says:-*

*“We are interested in improving the quality of health care in this country”*

*You might need to probe:*

*“Would it be helpful to have more evidence about what factors affect the quality of health care?”*

*Or if the policy maker says, we are wondering whether it is worthwhile investing in a new cadre of community health workers. You might probe:*

*“Would you be interested in having an evaluation done of the existing community health worker programme?”*

1. When you need to get research, evidence or advice about a particular health systems issue, who do you typically go to, to get this?

*If they DON’T say “the School of Public Health” (and if you are brave) you might ask why they don’t come to you.*

*7. Any additional comments or thoughts?*

*End of Component D*

## *Annex 1 Consent form for self administered questionnaires*

Iam -------------------- and Iam working with the ………..School of Public Health. We are doing needs assessment to assess the capacity of the ……………..School of Public health to undertake health systems research. The capacity assessment will involve review of documents, key informant interviews and self administered questionnaires. We shall also hold workshops to discuss these results. The results of the capacity assessment will inform the preparation of a capacity development plan which will be implemented by the school and supported by the Future Health Systems Research Program Consortium. We expect that the execution of this plan will enhance the capacity of the school to undertake health systems research.

For this particular part of the study, you have been identified as a critical and valued member whose opinions would assist in this assessment. We are therefore requesting you to complete a self administered questionnaire. There are no risks in your participation in this study. Please be assured that your name will be kept anonymous. You are free to refuse to participate. If you do agree to participate, we kindly request that you answer all the questions but you are free to skip any question that you prefer not to answer. You are free to ask me any questions about the study. Should you wish to contact us, you can do so at the address below.

I request you to confirmyour consent for the study by signing in the space below. I shall provide my signature as a witness to this. WE will both receive a copy each for our records.

Interviewer s signature ------------------------------------------------

Interviewee’s signature --------------------------------------------------

Date ------------------------- Place -----------------------------------------------------

Dr ....................................... ( Lead researcher )

...............School of Public Health

P.O.Box ...........

.............( City)........... ( Country),

### Annex 2: *Consent form for key informants for health systems capacity assessment*

Iam -------------------- and I am working with the …………..School of Public Health. We are doing needs assessment to assess the capacity of the ………….School of Public health to undertake health systems research. The capacity assessment will involve a review of documents, key informant interviews and self administered questionnaires. We shall also hold workshops to discuss these results to which we would welcome your attendance.

The results of the capacity assessment will inform the preparation of a capacity development plan which will be implemented by the school and supported by Future Health Systems Research Program Consortium.. We expect that the execution of this plan will enhance the capacity of the school to undertake health systems research.

For this particular part of the study, you have been identified as a critical and valued member whose opinions would assist in this assessment. We are therefore requesting you to serve as a key informant for the capacity assessment. There are no risks in your participation in this study. Please be reassured that your name will be kept anonymous. You are free to refuse to participate. If you do agree to participate you are free to refuse to answer any question that I may ask you in the course of the interview. You are free to ask me any questions about the study. Should you wish to contact us, you can do so at the address below.

Please note that this consent also allows us to record the interview in the event that we need to refer back to some of your responses and to ensure that you are quoted accurately. This will ensure the integrity of the interview and the accuracy of any transcription or quotation. Feel free to let me know if there are any responses that you would be prefer be kept off the record.

I request you to confirmyour consent for the study by signing in the space below. I shall provide my signature as a witness to this. We will both receive a copy each for our records.

Interviewer s signature ------------------------------------------------

Interviewee’s signature --------------------------------------------------

Date ------------------------- Place -----------------------------------------------------

Dr ....................................... ( Lead researcher )

...............School of Public Health

P.O.Box ...........

.............( City)........... ( Country),

**References**

1. Lusthaus, C., G. Anderson, and E. Murphy, *Institutional Assessment: A framework for strengthening capacity for IDRC's research partners*. 1995, Ottawa: International Development Research Center.

2. Kothari, A., et al., *Is research working for you? Validating a tool to examine the capacity of health organizations to use research.* Implement Sci, 2009. **4**: p. 46.

3. Bennett, S. and A. Corluka, *Health policy analysis institutes: Landscaping and learning from experience*. 2010, Alliance for Health Policy and Systems Research: Geneva.

4. Bennett, S., et al., *From Mexico to Mali: progress in health policy and systems research.* Lancet, 2008. **372**(9649): p. 1571-8.
